# Annexin II Light Chain p11 Interacts With ENaC to Increase Functional Activity at the Membrane

**DOI:** 10.3389/fphys.2019.00007

**Published:** 2019-02-08

**Authors:** Tanya T. Cheung, Noor A. S. Ismail, Rachel Moir, Nikhil Arora, Fiona J. McDonald, Steven B. Condliffe

**Affiliations:** ^1^Department of Physiology, University of Otago, Dunedin, New Zealand; ^2^Biochemistry Department, Faculty of Medicine, Universiti Kebangsaan Malaysia, Kuala Lumpur, Malaysia

**Keywords:** epithelial, sodium, channel, p11, annexin II, protein interaction, Na^+^ absorption, exocytosis

## Abstract

The epithelial Na^+^ channel (ENaC) provides for Na^+^ absorption in various types of epithelia including the kidney, lung, and colon where ENaC is localized to the apical membrane to enable Na^+^ entry into the cell. The degree of Na^+^ entry via ENaC largely depends on the number of active channels localized to the cell membrane, and is tightly controlled by interactions with ubiquitin ligases, kinases, and G-proteins. While regulation of ENaC endocytosis has been well-studied, relatively little is understood of the proteins that govern ENaC exocytosis. We hypothesized that the annexin II light chain, p11, could participate in the transport of ENaC along the exocytic pathway. Our results demonstrate that all three ENaC channel subunits interacted with p11 in an *in vitro* binding assay. Furthermore, p11 was able to immunoprecipitate ENaC in epithelial cells. Quantitative mass spectrometry of affinity-purified ENaC-p11 complexes recovered several other trafficking proteins including HSP-90 and annexin A6. We also report that p11 exhibits a robust protein expression in cortical collecting duct epithelial cells. However, the expression of p11 in these cells was not influenced by either short-term or long-term exposure to aldosterone. To determine whether the p11 interaction affected ENaC function, we measured amiloride sensitive Na^+^ currents in *Xenopus* oocytes or mammalian epithelia co-expressing ENaC and p11 or a siRNA to p11. Results from these experiments showed that p11 significantly augmented ENaC current, whereas knockdown of p11 decreased current. Further, knockdown of p11 reduced ENaC cell surface population suggesting p11 promotes membrane insertion of ENaC. Overall, our findings reveal a novel protein interaction that controls the number of ENaC channels inserted at the membrane via the exocytic pathway.

## Introduction

Whole body Na^+^ balance primarily depends on the co-ordinated actions of multiple ion transport proteins in the apical and basolateral membranes of renal epithelial cells (Rossier et al., 2015). Being localized to the apical membrane of late distal tubule and cortical collecting duct (CCD) cells, the epithelial Na^+^ channel (ENaC) allows for Na^+^ entry thereby representing the rate-limiting step for transepithelial Na^+^ absorption (Hager et al., 2001; Pearce et al., 2015). ENaC is composed of three subunits (α, β, and γ) arranged in a heterotrimeric stoichiometry (Noreng et al., 2018). Each subunit is comprised of a large extracellular loop, two transmembrane domains and short cytoplasmic N- and C-terminal domains. Biophysically, ENaC is characterized by having a small single channel conductance, high selectivity for Na^+^ over K^+^ (Kellenberger et al., 1999) and an average open probability (Po) of 0.4–0.5 that depends on several factors including the degree of proteolytic cleavage of ENaC extracellular domains (Sheng et al., 2006), ENaC phosphorylation (Bao et al., 2014), mechanical forces (Althaus et al., 2007) and sodium concentration (Hanukoglu and Hanukoglu, 2016; Hanukoglu, 2017).

Given its essential role in Na^+^ homeostasis, ENaC function is highly regulated by several hormones, particularly by aldosterone (Rossier et al., 2015). The major effect of aldosterone on ENaC results in an increase in the number of ENaC channels in the apical membrane. This mainly occurs via activation of late response genes that increase α-ENaC subunit synthesis (Masilamani et al., 1999) and early response genes that restrict endocytosis of ENaC (Snyder et al., 2002; Soundararajan et al., 2005). For example, serum- and glucocorticoid-regulated kinase 1 (SGK1) expression is rapidly increased by aldosterone which, when activated, phosphorylates neural precursor cell-expressed developmentally downregulated gene 4-2 (*Nedd4-2*) preventing this ligase from ubiquitinating ENaC, thereby inhibiting ENaC endocytosis and subsequent degradation (Snyder et al., 2002). In contrast, the effects of aldosterone on ENaC exocytosis and the signaling proteins involved are not as well-understood. Constitutive exocytic trafficking of ENaC from the Golgi to the membrane depends on multiple trafficking machineries (Goldfarb et al., 2006; Hill et al., 2007; Pochynyuk et al., 2007) while the final vesicle fusion events controlling ENaC membrane insertion involve SNAREs (Condliffe et al., 2003, 2004) and accessory proteins (Butterworth et al., 2005). Signaling proteins that have been shown to modulate ENaC exocytic trafficking include the Rab-GAP AS160 (Liang et al., 2010), Rab11b (Butterworth et al., 2012), Ankyrin G (Klemens et al., 2017), and protein kinase D (Dooley et al., 2013).

The p11 protein, also known as S100A10 or annexin II light chain, has a broad tissue distribution with highest expression found in brain, kidney, lung, and intestine (Saris et al., 1987; O’Kelly and Goldstein, 2008). Cellular localization is mainly restricted to the cytosol where it forms a heterotetrameric complex with the Ca^2+^/lipid binding protein, annexin II/annexin A2. This association is important for targeting the complex to sites of exocytosis on the cytosolic face of the plasma membrane (Thiel et al., 1992; Umbrecht-Jenck et al., 2010). In addition to annexin II, p11 has been shown to interact with the cytosolic domains of a number of ion channels. This was first described for the Na_V_1.8 channel where p11 binding to the amino terminal domain promotes the translocation of the channel to the plasma membrane (Okuse et al., 2002). Similar effects of p11 have been observed with the TASK-1 K^+^ channel (Girard et al., 2002) and acid-sensing ion channel ASIC1a (Donier et al., 2005). Interestingly, p11 interactions are also required for targeting TRPV5, TRPV6 (van de Graaf et al., 2003) and CFTR channels (Borthwick et al., 2007) to the apical membrane in epithelial cells. These observations have led to the proposal of a model where p11 binding directs the exocytic trafficking of ion channels to the plasma membrane (Rescher and Gerke, 2008).

Since the cellular mechanisms that regulate ENaC trafficking along the exocytic pathway are relatively unclear and p11 has been shown to play an important role in this process, we hypothesized that p11 could interact with ENaC in the exocytic pathway to target the channel to the plasma membrane. To test this hypothesis, we initially used *in vitro* binding and co-immunoprecipitation techniques to probe for a physical interaction between ENaC channel subunits and p11. This interaction was further evaluated using a mass spectrometry proteomics approach. We also examined the endogenous level of p11 protein expression and its potential regulation by aldosterone in CCD cell lines. In addition, possible effects of p11 on ENaC function were measured using two-electrode voltage clamp electrophysiology in *Xenopus laevis* oocytes, and short-circuit current in epithelial monolayers. Our results indicate that p11 binds to ENaC and this interaction is associated with alterations in ENaC whole cell current, and transepithelial amiloride-sensitive current.

## Materials and Methods

### Cell Culture and Transfection

Cell lines were imported under and transfection was approved under Environmental Protection Authority (EPA) approvals APP201858 and APP201859 respectively. All cell lines were grown in a humidified atmosphere of 5% CO_2_ at 37°C. HEK293 and COS-7 cells were maintained in DMEM with 10% FBS, 1% penicillin/streptomycin and 1% glutamine (all Life Technologies, from Thermo Fisher Scientific, Auckland, New Zealand). M1 mouse CCD cells were maintained in DMEM:Ham’s F12 medium (without phenol red) supplemented with 10% FBS, 1% Glutamax, 1% penicillin/streptomycin and 1 mM dexamethasone (Life Technologies). Fischer rat thyroid (FRT) cells were maintained in Ham’s F12 (Sigma, New Zealand) supplemented with 10% FBS and 1% penicillin/streptomycin. Mouse CCD clone 1 (mCCD_cl1_) cells were maintained in a defined medium as described in Gaeggeler et al. (2005). For experiments investigating the effect of aldosterone on p11 expression, M1 or mCCD_cl1_ cells were grown to 80% confluency whereupon normal growth medium was replaced with serum and dexamethasone free medium. After 24 h, aldosterone (Sigma, to a final concentration of 10 nM) or a vehicle control (ethanol) was added to M1 or mCCD_cl1_ cells for 1, 3, or 24 h. Cells were then lysed for immunoblotting as described below. ENaC plasmids, including HA (hemagglutinin, YPYDVPDYA) epitope tagged versions, were described previously (McDonald et al., 1995; Wiemuth et al., 2007; Fronius et al., 2010). FRT, HEK293 and COS-7 cells were transfected with either HA-tagged or wild type α-, β-, and γ-ENaC subunits using Lipofectamine 2000 (Thermo Fisher Scientific) or CaCl_2_ precipitation methods.

### Pull-Down Assays, Immunoblotting, and Co-immunoprecipitation

Recombinant DNA experiments were conducted under EPA approval APP201859. Glutathione *S*-transferase (GST) pull-down assays were performed as described previously (Wiemuth et al., 2007). Briefly, p11 proteins tagged with GST (O’Kelly and Goldstein, 2008), or GST alone were produced in BL21 *E. coli* and purified on glutathione-agarose beads (Sigma, Auckland, New Zealand). COS-7 cell lysates containing HA-tagged α-, β-, or γ-ENaC were precleared for 1 h at 4°C with GST bound to glutathione-agarose beads. Cleared lysates were then incubated with 50 μg of GST-p11 or GST alone for 3 h at 4°C. The glutathione-agarose beads, fusion proteins and any interacting proteins were collected by centrifugation. After extensive washing with 1% Triton in PBS buffer, samples were resolved on a 8% SDS-PAGE gel, transferred to PVDF membrane (Roche Biochemicals, distributed by Sigma New Zealand) at 45 mA for 2 h (Hoefer semi-dry transfer). Membranes were probed with anti-HA antibodies (H6908, Sigma New Zealand, 1:1000) applied in blocking buffer (20 mM Tris, pH 7.4, 150 mM NaCl, 0.1% Tween 20, all Sigma) and 5% non-fat dry milk (Fonterra, New Zealand). The secondary antibody HRP-conjugated goat anti-rabbit (A6154, Sigma) was used at 1:10,000 dilution.

To evaluate endogenous p11 and annexin II protein expression in M1 or mCCD_cl1_ cells, cells were solubilized in lysis buffer (138 mM NaCl, 20 mM Tris-HCl, 1% Triton X-100, 10 mg/ml PMSF and 3 μg/ml aprotinin, pH 7.4, all from Sigma) and protein concentration evaluated according to Bradford’s protocol (Bio-Rad Laboratories, Auckland, New Zealand). Protein samples were loaded onto a 15% SDS-PAGE and transferred onto PVDF membrane as above. The primary antibodies used were: rabbit anti-p11 at 1:1000 (ab76472, Abcam, Melbourne, VIC, Australia), or mouse anti-p11 at 1:1000 (ab89438, Abcam) and rabbit annexin II 1:5000 (41803, Abcam), overnight at 4°C.

For co-immunoprecipitation, HEK293 cells were co-transfected with p11-myc-DDK plasmid (RC204992, OriGene Technologies, Rockville, MD, United States) and plasmids encoding α-, β_HA_-, and γ-ENaC subunits. After 24 h, cells were lysed in 1% Triton in 1x TBS (50 mM Tris-HCl, 150 mM NaCl, pH 7.4–7.5) with protease inhibitors. Mouse anti-p11 antibody was used to immunoprecipitate p11 along with protein G-agarose beads. The isolated immuno-protein complex was then washed and used for Western blot analysis (12% SDS-PAGE) with anti-HA and anti-p11.

### Surface Biotinylation Assay

Fischer rat thyroid cells were co-transfected with 1 μg each of plasmids encoding α-, β_HA,_ or γ-ENaC with 20 pmol control or p11 siRNA (Sigma-Aldrich, St. Louis, MO, United States). Cells were washed five times in ice-cold PBS 48 h post-transfection. Surface proteins were labeled as described in Butterworth et al. (2005). Briefly, cells were biotinylated in borate buffer (85 mM NaCl, 4 mM KCl, 15 mM Na_2_B_4_O_7_, 10 μg/ml PMSF, 1 μg/ml pepstatin, 2 μg/ml leupeptin, 2 μg/ml aprotinin pH 9.0) with 1 mg/mL Sulfo-NHS-LC-Biotin (Life Technologies New Zealand Limited) for 20 min on ice with shaking. After removal of biotin, signal was quenched with PBS containing 10% FBS. Cells were washed again five times in ice-cold PBS and lysed in biotinylation lysis buffer (50 mM EDTA, 10 mM Tris, 1% NP-40, 0.4% sodium deoxycholate, pH 7.4) for 10 min on ice while shaking. Lysates were transferred to a 1.5 mL tube and centrifuged for 10 min at 16.1 rcf at 4°C. To equal amounts of proteins (as determined by Bradford assay), 100 μL NeutrAvidin (Pierce^TM^NeutrAvidin^TM^Agarose, Life Technologies New Zealand Limited) beads were added and incubated overnight at 4°C with rotation. Following incubation, samples were centrifuged at 0.4 rcf to pellet beads (surface), while supernatant (cytosolic) fractions were transferred to new tubes. Beads were washed four times with biotinylation lysis buffer, with centrifugation at 0.4 rcf and then boiled at 95°C for 5 min. Samples were then separated by 8% SDS-PAGE gel followed by immunoblot analysis.

### Mass Spectrophotometry Analysis

HEK293 cells were co-transfected with p11-myc-DDK plasmid and plasmids encoding α-, β_HA_-, and γ-ENaC subunits as above. A first round of immunoprecipitation was performed using either anti-HA antibody to immunoprecipitate βENaC-HA or anti-DDK (TA50011, OriGene Technologies) to immunoprecipitate p11-myc-DDK protein from the pre-cleared lysate. The isolated immuno-protein complex was then washed and subjected to a second round of immunoprecipitation using anti-DDK or anti-HA to isolate proteins associated with the βENaC-p11 complex from the first round of immunoprecipitation. The immune-protein complexes were then divided into two tubes in equal amounts, one was used for Western blot analysis (12% SDS-PAGE) with anti-HA and anti-myc DDK (TA50011, OriGene, United States) to confirm β-ENaC_HA_ and p11 expression respectively, while the other was processed for mass spectrometry (Figure 3A). Co-immunoprecipitated p11, ENaC and other interacting proteins were denatured in SDS-PAGE sample buffer, and proteins were separated on individual 12% SDS-PAGE gels using standard techniques. The gels were stained with Coomassie G250 and protein lanes cut into three fragments for liquid chromatography coupled with electrospray ionization linear ion trap [LC-ESI LTQ] Orbitrap tandem mass spectrometry [MS/MS]. Fractions were subjected to in-gel protein digestion with trypsin as described previously (Shevchenko et al., 1996). Each digested fraction was concentrated using a centrifugal vacuum concentrator and reconstituted in a 15 μl aqueous solution of 2% (vol/vol) acetonitrile (ACN) supplemented with 0.2% formic acid for LC-ESI LTQ Orbitrap analyses.

Peak lists were processed through the Proteome Discoverer 1.1 software (Thermo Scientific, San Jose, CA, United States) for all ESI LTQ Orbitrap data using the software’s default settings. All peak lists were then searched with an in-house Mascot server (version 2.1.0; Matrix Science) against an amino acid sequence database combining all predicted and translated p11 and all entries from the NCBI non-redundant sequence database, matching the taxa *Homo sapiens*. Mascot search settings allowed for full tryptic peptides with up to three missed cleavage sites and variable modifications of carbamidomethyl (C) and oxidation (M). The precursor and fragment mass tolerances were set to 10 ppm and 0.8 Da. To evaluate the false-discovery rate (FDR), all peak lists were searched against a decoy database using identical search settings. The decoy database was built using the decoy database tool at the Trans-Proteomic Pipeline (TPP; Seattle Proteome Center), comprising the reversed sequence entries of the aforementioned combined database. The FDR was calculated by determining the number of false-positive peptide hits from the decoy search versus the number of peptide identifications from the true search using the same Mascot score as a significance threshold.

Only peptide hits with an individual ion score of >25 (Mascot significance threshold at a *p* of < 0.05) were accepted as significant identifications. This resulted in an FDR of <0.02 for all searches. Significant protein identification required at least two significant peptide hits covering different sequences of the protein. In addition, a protein that was identified by single peptide-based protein identification in one experiment was also confirmed by a different peptide identification covering another sequence stretch in one of the other experiments was considered a significant multipeptide identification.

### qRT-PCR of p11 and Annexin II

mCCD_cl1_ cells were exposed to aldosterone or vehicle as above. Cells were lysed in Trizol (Life Technologies) and RNA was isolated according to manufacturer’s instructions. cDNA was prepared using the PrimeScript RT Reagent Kit (Perfect Real time, Medi’Ray, New Zealand) and qPCR was carried out with SYBR Premix Ex Taq (Tli Rnase H Plus) ROX plus (Medi’Ray, New Zealand). KiCqStart^TM^ primers for *18S* (Fwd 5′-CAGTTATGGTTCCTTTGGTC-3′, Rev 5′-TTATCTAGAGTCACCAAGCC-3′), *p11* (Fwd 5′-GGAAAATCAAAAGGACCCTC-3′, Rev 5′-CCTTCTGCTTCATGTTTACTAC-3′), *Annexin II* (Fwd 5′-CTCCAGAAAGTGTTCGAAAG-3′, Rev 5′-TTCTAATCAGGACCTTGTCTC-3′), and *Sgk1* (Fwd 5′-ATCCTGAAGAAGAAAGAGGAG-3′, 5′-ATGTAGTCCAGGACAAAGTAG-3′) were from Sigma. Reactions were analyzed on a CFX Connect PCR machine (Bio-Rad). Analysis of gene expression was calculated using the following equation: ΔCt = 2^-(Ctgene-Ctreference)^.

### Heterologous Expression in *Xenopus* Oocytes and Two-Electrode Voltage Clamp Electrophysiology

cDNAs of human ENaC subunits and human p11 (p11-myc-DDK) were linearized and cRNAs synthesized *in vitro* with a T7 mMESSAGE mMACHINE^TM^ transcription kit (Ambion, from Thermo Fisher Scientific). Defolliculated oocytes (Stages V–VI) isolated from *Xenopus laevis* in accordance with guidelines approved by The University of Otago Animal Ethics Committee were injected with 0.75 ng of cRNA of each ENaC subunit. For experiments investigating the effect of p11 co-expression on ENaC function, oocytes were co-injected with 0.75 ng of p11 cRNA. After injection, oocytes were maintained at 18°C in low Na^+^ modified Barth’s solution [83 mM NMDG, 5 mM NaCl, 1 mM KCl, 0.33 mM Ca(NO_3_), 0.41 mM CaCl_2_, 10 mM HEPES, and 1% penicillin/streptomycin; pH 7.4. all from Sigma] for 1–2 days before ENaC recordings.

Two-electrode voltage clamp recordings were performed on ENaC-expressing oocytes using microelectrodes filled with 1 M KCl connected to a Turbo TEC 05 amplifier (NPI, Tamm, Germany). Oocytes were bathed and continuously perfused in ND96 solution (96 mM NaCl, 1 mM KCl, 1.8 mM CaCl_2_, 1 mM MgCl_2_, and 5 mM HEPES, pH 7.4, all Sigma) After impalement, membrane potentials were allowed to stabilize before voltage clamping at -60 mV. Amiloride-sensitive, ENaC specific Na^+^ currents were then recorded as the difference in current before and after the addition of amiloride (10 mM final concentration, Tokyo Chemical Industry, from Lab Supply, Dunedin, New Zealand).

### Transepithelial Ion Current Measurements

Fischer rat thyroid cells were seeded onto 12 mm Snapwell^TM^ membranes (COR3801, Corning via In Vitro Technologies, New Zealand) at a density of 4 × 10^5^ cells per Snapwell^TM^ and incubated overnight. Cells were transfected with 0.067 μg of plasmids encoding *α-, β-*, or *gENaC* and 20 pmol siRNA targeting *p11*, or control siRNA, using Lipofectamine^TM^ 3000 (Invitrogen). The transfection medium was replaced with FRT full-growth media supplemented with 10 μM amiloride 6 h post-transfection. After 72 h, epithelia were mounted onto a modified Ussing chamber connected to a multichannel V/A clamp (Physiologic Instruments, San Diego, CA, United States) via a DI-720 data acquisition system (DataQ Instruments). Data was recorded using the Acquire and Analyze 2.3 program (Physiologic Instruments) running on a PC. The apical and basolateral surfaces were bathed in 1x Ringer’s solution (in mM 135 NaCl, 2.4 K_2_HPO_4_, 10 HEPES, 1.2 CaCl_2_, 1.2 MgCl_2_, pH 7.4–7.5), kept at 37°C and bubbled with O_2_. The epithelia were clamped under short-circuit conditions and the amiloride-sensitive short circuit current (*I*_sc_-amil) was measured. The *I*_sc_-amil was determined as the difference in current recorded prior to and after the addition of amiloride (5 μM final concentration) into the apical bathing solution. The relative *I*_sc_-amiloride was obtained by normalizing the *I*_sc_-amiloride to that of cells transfected with *αβγ ENaC* alone in a parallel experiment. Transepithelial resistance (*R*t) was monitored by applying repetitive 5 mV pulses for 1 s at 120 s intervals.

### Data Analysis

All data are expressed as the mean ± SEM of *n* experiments. Statistical significance between two groups was determined using an unpaired *t*-test or one-sample *t*-test at the *p* level indicated. ANOVA was used for multiple comparisons with a *post hoc* Bonferroni’s test to identify significant differences between groups at the *p* level indicated. Data was analyzed for normal distribution using the D’Agostino-Pearson normality test on Prism 7 (GraphPad Software, United States).

## Results

### p11 Interacts With ENaC Subunits

**FIGURE 1:**
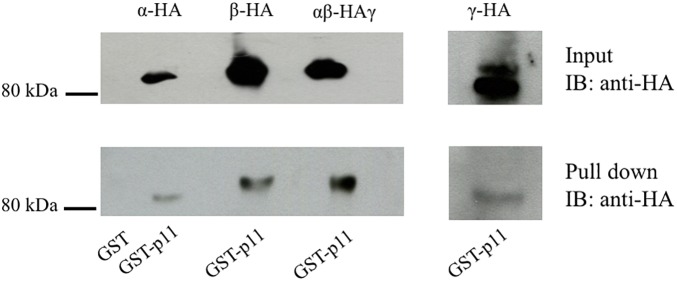
p11 interacts with all three epithelial Na^+^ channel (ENaC) subunits. HA-tagged ENaC subunits were transiently expressed alone or together as an αβ_HA_γ channel complex in COS-7 cells. Cells were then lysed, pre-cleared with GST bound to glutathione-agarose beads and then incubated with 50 μg of GST alone or GST-p11. After washing, bound proteins were eluted and analyzed by SDS-PAGE and immnoblotting (IB) with anti-HA antibody. The top panel labeled ‘Input’ shows the successful expression of each subunit (Mr 85 kDa). The bottom lane labeled ‘Pulldown’ shows binding of each ENaC subunit and the channel complex to GST-p11. The GST lane indicates that no ENaC subunits interacted with GST alone. Data shown are representative of three independent experiments with similar results.

Both the activity of ENaC at the membrane and the number of membrane resident channels are influenced by multiple regulatory proteins directly interacting with ENaC channel subunits (Snyder et al., 2002; Biasio et al., 2004; Condliffe et al., 2004; Butterworth, 2010). Since p11 interacts with several apical membrane proteins (van de Graaf et al., 2003; Borthwick et al., 2007), we investigated whether p11 directly binds to ENaC channel subunits. A GST-p11 protein was used in pull-down experiments with HA-tagged α-, β-, or γ-ENaC subunits. As shown in the lower panel of Figure 1, GST-p11 was able to pull-down all three ENaC subunits either as individual subunits or as a trimeric complex of α-, β_HA_-, and γ-ENaC. In contrast, no interaction was detected between GST alone and ENaC (Figure 1, first lane, and Supplementary Figure S1). These results demonstrate that p11 is capable of binding to each ENaC subunit either as a single protein or to the heteromultimeric ENaC complex.

**FIGURE 2:**
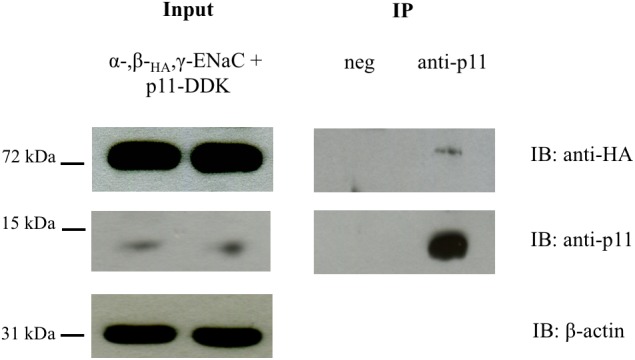
p11 co-immunoprecipitates with ENaC. HEK293 cells were transfected with p11-myc-DDK and αβ_HA_γ ENaC. After 24 h, cells were lysed and immunoprecipitated with anti-p11 (ab89438) and protein G-agarose beads. Precipitates were analyzed by SDS-PAGE and immunoblotted (IB) with anti-HA to detect β_HA_ ENaC (Mr ∼ 72 kDa) and p11 (Mr ∼ 11 kDa). ENaC was co-immunoprecipitated in the presence of anti-p11 but not when an unrelated antibody was used. Data shown are representative of three independent experiments with similar results.

To further evaluate the interaction between ENaC and p11 and to confirm that this occurred in intact cells, HEK293 cells were co-transfected with p11-myc-DDK and α-, β_HA_-, and γ-ENaC. Complexes between p11-myc-DDK and ENaC were immunoprecipitated with an anti-p11 antibody and protein G-agarose beads before being analyzed by Western blotting using an anti-HA antibody. In control samples where only p11 was expressed and protein precipitated using a mouse p11 antibody, no ENaC protein was detected by immunoblot using the anti-HA antibody. In contrast, in samples co-expressing p11 and ENaC, p11 was able to co-immunoprecipitate bENaC which was revealed by immunoblotting by anti-HA antibody (Figure 2 and Supplementary Figure S2). This data indicates that p11 and ENaC are able to interact in mammalian cells, in support of our *in vitro* pull-down results.

### Identification of Novel p11-ENaC Interacting Partners

**FIGURE 3:**
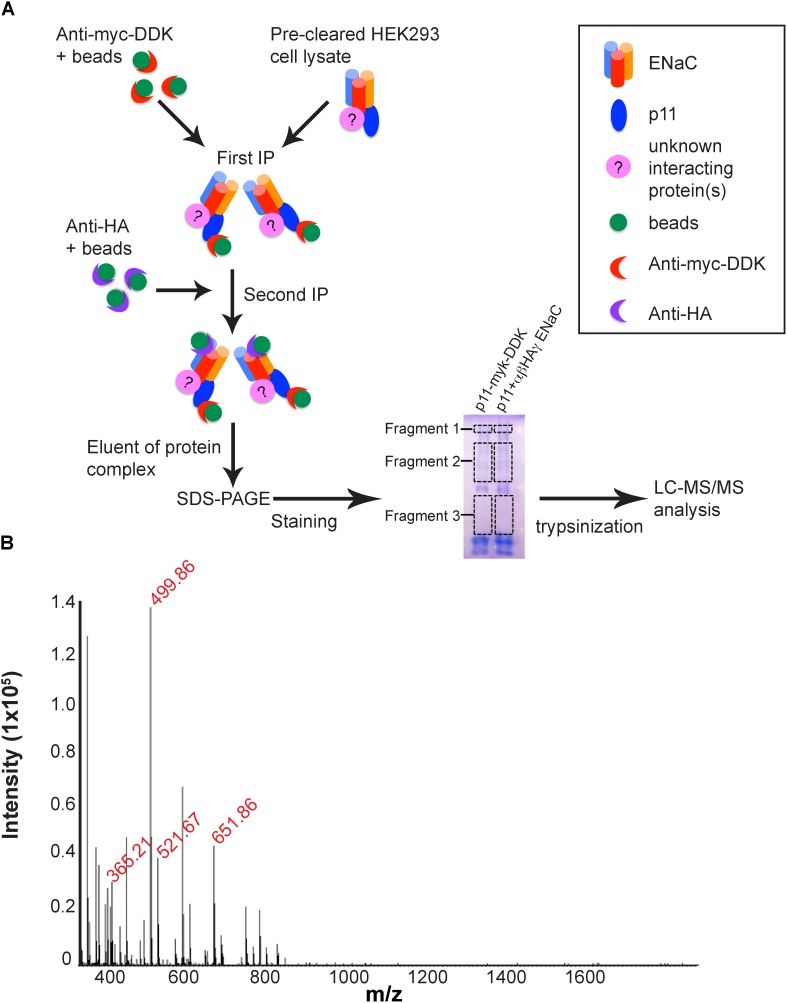
Identification of novel p11-ENaC interacting partners by LC–MS/MS analysis. **(A)** Schematic representation of the proteomics approach used to identify proteins interacting with the p11-ENaC complex. HEK293 cell lysates were co-transfected with p11-myc-DDK and αβ-HAγ ENaC. After 24 h expression, cells were lysed and co-immunoprecipitated sequentially with anti-myc-DDK and anti-HA with additional protein G-agarose beads in each tube. The eluent of the complex was then collected and subjected to SDS-PAGE 1D gel. The gel was carefully excised into three fragments and processed through in-gel trypsinization for LC–MS/MS analysis to determine potential proteins interacting with ENaC and p11. **(B)** MS spectrum recorded during analysis of purified protein complexes. Red text denotes specific peptides that identified proteins listed in Table 1. The list of identified peptides was used to tag the corresponding peptide-related ion spectra based on m/z differences, deviations from the predicted elution times, and the match between the theoretical and observed isotopic envelopes. The maximum deviation accepted in m/z and the retention time was established separately for each of the processed LC–MS spectra to account for possible variations in mass measurement accuracy and chromatographic separation between runs.

In order to identify putative interaction partners of the p11-ENaC complex, the immunopurified fraction of HEK293 lysates transfected with tagged ENaC and p11 constructs were isolated by gel electrophoresis, in-gel trypsinized and the resulting peptide fragments analyzed by mass spectroscopy (Figure 3A). Table 1 lists the proteins identified by mass spectrometric sequencing of MS spectrum peaks (Figure 3B) that are known to have roles in protein trafficking pathways trafficking pathways, and Supplementary Table S1 lists all proteins identified. Several tubulin proteins of the microtubule network required for transporting vesicles to the plasma membrane were detected with high Mascot and unique peptide scores. In addition, HSP-90 and annexin A6 proteins involved in exocytic trafficking associate with the p11-ENaC complex. Interestingly, the mass spectrometry analysis also identified the E3 ubiquitin protein ligase RNF2 protein confirmed to play a role in ubiquitination pathways (Xia et al., 2014) suggesting that the p11-ENaC complex persists at the plasma membrane before being targeted for endocytosis and subsequent recycling or degradation. Altogether, these results confirm that the p11-ENaC complex is able to interact with multiple proteins involved in controlling ENaC trafficking to and from the plasma membrane.

**Table 1:** Trafficking proteins identified by LC–MS/MS analysis.

Protein	Gene name	Accession number	Mascot score	Role
				
β -Tubulin	*TUBB4B TUBB2A*	P68371 Q13885	149.74 134.90	Cytoskeletal component
α -Tubulin	*TUBA1C*	Q9BQE3	119.45	Cytoskeletal component
Heat shock protein-90	*HS90B*	P08238	78.99	Protein folding
Ring finger protein-2	*RNF2*	Q99496	24.52	E3 ubiquitin protein ligase
Annexin A6	*ANXA6*	P08133	3.51	Membrane binding protein


*Proteins with roles in intracellular trafficking identified by mass spectrometry sequencing of material precipitated with the p11-ENaC complex and associated Mascot score.*

### Expression of Endogenous p11 in Mouse CCD Cells

**FIGURE 4:**
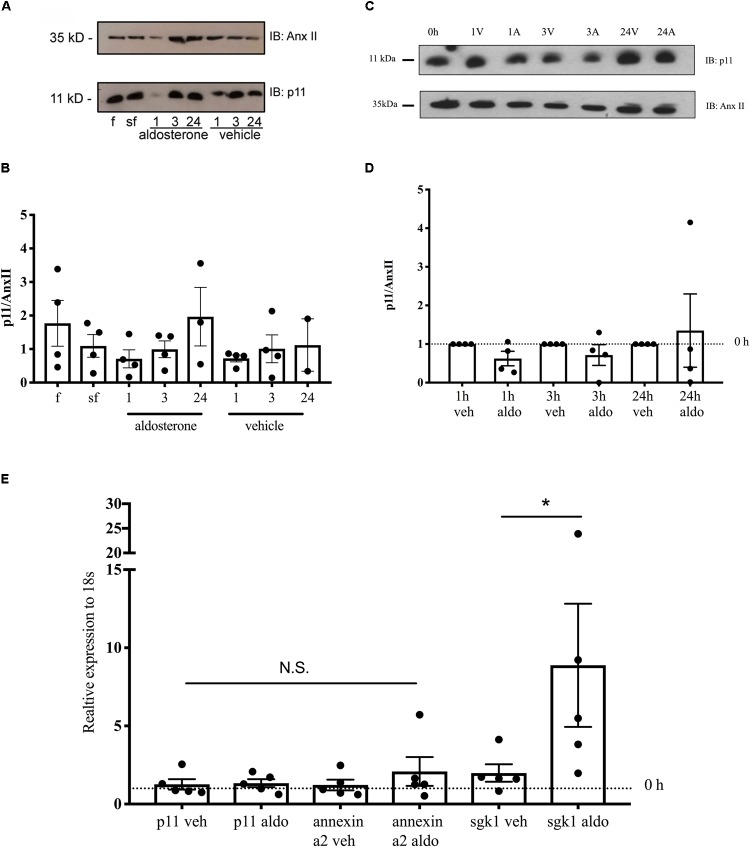
Effect of aldosterone on the endogenous expression of p11. M1 CCD or mCCD_cl1_ cells were grown to 80% confluence in full medium (F) then 24 h in dexamethasone and serum-free (SF) medium before treatment with 10 nM aldosterone or ethanol (veh) for 1, 3, or 24 h. Cells were then lysed and immunoblotted (IB) for p11 subunit (Mr ∼ 11 kDa) and annexin II (Anx II or annexin a2) subunit (38 kDa), or used for qRT-PCR. **(A,C)** Representative western blot showing the presence of endogenous p11 and annexin II proteins in M1-CCD cells and the effects of aldosterone versus vehicle treatment. **(B,D)** Quantification of p11 protein level normalized to annexin II in M1 CCD or mCCD_cl1_ cells in culture conditions described above. **(E)** *p11, Annexin II*, and *Sgk1* expression is shown relative to expression of *18S* following 1 h of 10 nM aldosterone treatment. The threshold value was automatically set by the CFX Manager software and the Ct value was calculated from the average of three technical replicates. Data are expressed as mean ± SEM (*n* = 3–4 independent experiments) by one-way ANOVA followed by Bonferroni’s post-test where ^∗^ indicates a significant difference to control at a level of *p* < 0.05.

The epithelial Na^+^ channel has a pivotal role in Na^+^ reabsorption of the CCD of the kidney where its expression is upregulated by aldosterone. To verify whether CCD cells also endogenously express p11 and if aldosterone can increase this expression, we examined the level of p11 protein expression in the mouse M1-CCD cell line in the absence and presence of aldosterone. Under standard culture conditions, a robust expression of endogenous p11 protein was detected (Figure 4A and Supplementary Figure S3). Removal of serum from the media for 24 h resulted in p11 expression levels that were not significantly different from full medium (Figure 4A,B). To examine whether endogenous p11 expression was regulated by aldosterone, M1 cells were exposed to aldosterone (10 nM final concentration) or vehicle (ethanol) for 1, 3, or 24 h before quantifying p11 protein levels. Since typical housekeeping genes such as GAPDH and actin have been shown to be aldosterone sensitive in CCD cells (Perlewitz et al., 2010), we normalized p11 expression to the expression of annexin II which is not altered by aldosterone (Perlewitz et al., 2010). Similar to p11, annexin II was also endogenously expressed in M1 cells (Figure 4A) which is important given its role as a p11 interacting protein. Quantification of p11 protein level normalized to annexin A2 shows that p11 protein level was not significantly altered by short-term (1–3 h) aldosterone (10 nM) exposure, remaining at similar levels to serum free conditions (Figure 4B). However, incubating M1 cells in aldosterone (10 nM) for 24 h caused a higher p11 protein level to approximately the same level as observed in full medium although ANOVA did not detect a significant difference (Figure 4B). In contrast, treating cells with an ethanol vehicle for 24 h had no significant effect on p11 protein level (Figure 4B). These results demonstrate that CCD cells endogenously express p11 and annexin II, however aldosterone did not stimulate a significant increase in p11 protein synthesis.

As it is possible that the subclone of M1 cells used did not express the mineralocorticoid receptor (Náray-Fejes-Tóth et al., 2004), and because we found SGK1 was not markedly increased in the presence of aldosterone (data not shown), we repeated these experiments in mCCD_cl1_ cells to test if aldosterone altered protein levels of p11 and annexin II. mCCD_cl1_ cells were subjected to aldosterone (10 nM final concentration) or vehicle (ethanol) for 1, 3, or 24 h before quantifying p11 and annexin II protein levels using Western blot. Figure 4C,D and Supplementary Figure S4 shows that neither short or long term aldosterone treatment changed p11 or annexin II protein level. Finally, to test for a short term effect of aldosterone on p11 or annexin II mRNA (similar to the effect reported for SGK1, Náray-Fejes-Tóth et al., 1999), we subjected mCCD_cl1_ to 1 h aldosterone treatment, and quantified mRNA levels using qRT-PCR. Figure 4E shows that although SGK1 mRNA was increased after 1 h aldosterone treatment the mRNA levels of p11 and annexin II were not changed.

### Effect of p11 on ENaC Channel Function

**FIGURE 5:**
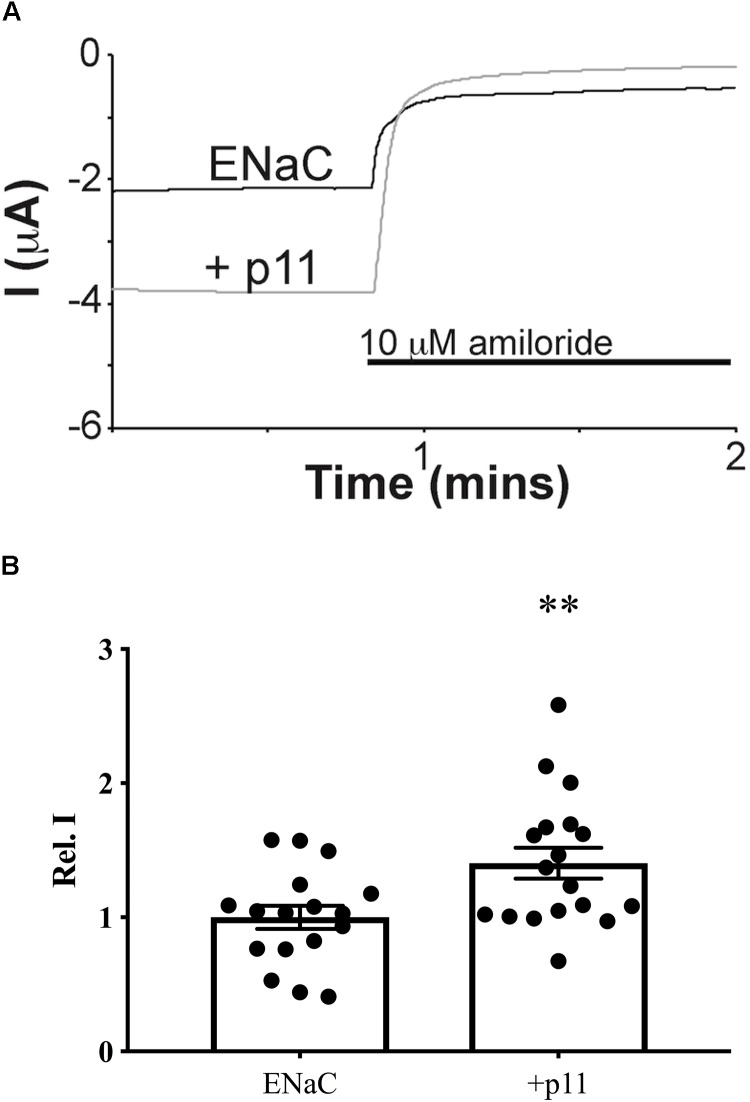
Epithelial Na^+^ channel current is increased by p11 co-expression. Oocytes were injected with α-, β-, and γ ENaC subunit cRNAs with or without p11 cRNA before recording whole cell Na^+^ currents using two electrode voltage clamp. **(A)** Representative whole cell Na^+^ currents from oocytes expressing ENaC alone (black line) or together with p11 (gray line) and the effect of perfusing 10 μM amiloride. **(B)** Relative amiloride-sensitive currents in oocytes injected with ENaC alone (*n* = 17) or together with p11 (*n* = 18). Oocytes were obtained from four different batches/animals. Amiloride-sensitive currents were measured as the difference before and after addition of 10 μM amiloride at a holding potential of –60 mV. Data are expressed as the mean (± SEM) fraction of amiloride-sensitive current from each oocyte relative to the ENaC control mean where ^∗∗^ indicates a significant difference to control at a level of *p* < 0.01 by unpaired *t*-test.

Having demonstrated that p11 physically interacts with ENaC, we next investigated whether p11 affected ENaC channel function by heterologous expression of p11 and ENaC in *Xenopus* oocytes. Amiloride-sensitive current was used to quantify the amount of ENaC-specific current using two electrode voltage clamp recordings of ENaC expressing oocytes. Figure 5A shows that both baseline current and the amount of amiloride-sensitive current were higher in oocytes co-expressing ENaC and p11 compared to oocytes expressing ENaC alone. On average, p11 co-expression caused a significant increase in relative amiloride-sensitive current at -60 mV from 1.0 ± 0.09 (*n* = 17) in oocytes injected with ENaC RNA alone to 1.4 ± 0.12 (*n* = 18) in oocytes injected with both ENaC and p11 RNA (Figure 5B). Water injected control oocytes had no significant amiloride-sensitive current (data not shown). These results suggest that, in addition to interacting with ENaC channels, p11 exerts a positive effect on ENaC channel function at the plasma membrane.

### Knockdown of p11 Reduces ENaC Current in Polarized Epithelia

**FIGURE 6:**
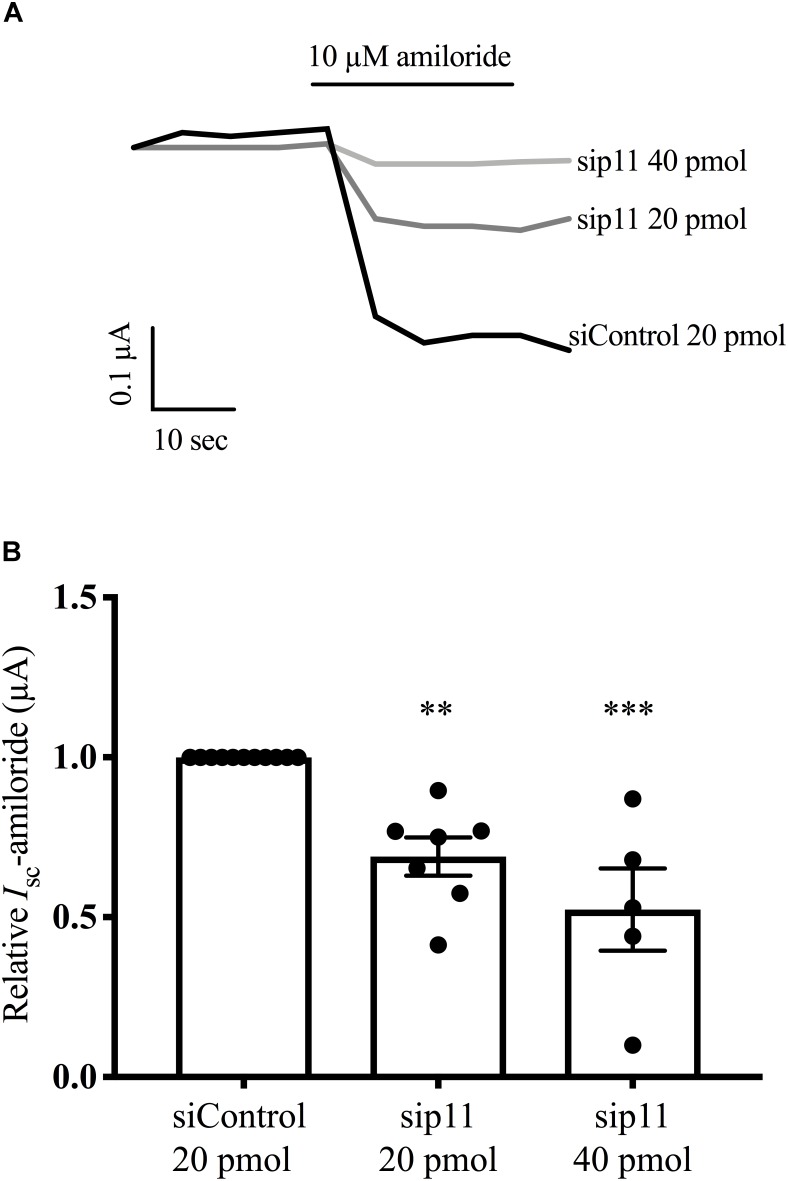
Knockdown of p11 reduces ENaC current. FRT epithelia were grown on Snapwell^TM^ filters, and co-transfected with plasmids encoding α-, β-, and γ ENaC (0.067 μg each) together with either 20 pmol of control siRNA or p11 siRNA. Amiloride-sensitive short-circuit current (*I*_sc_-amiloride) was measured 72 h after transfection. **(A)** Representative traces of *I*_sc_-amiloride for control and p11 knockdown epithelia. **(B)** Pooled results of *I*_sc_-amiloride for control and p11 knockdown epithelia, where ^∗∗^ indicates a significant difference to control at a level of *p* < 0.01 or ^∗∗∗^*p* < 0.001 by one sample *t*-test, *n* = 5–7.

**FIGURE 7:**
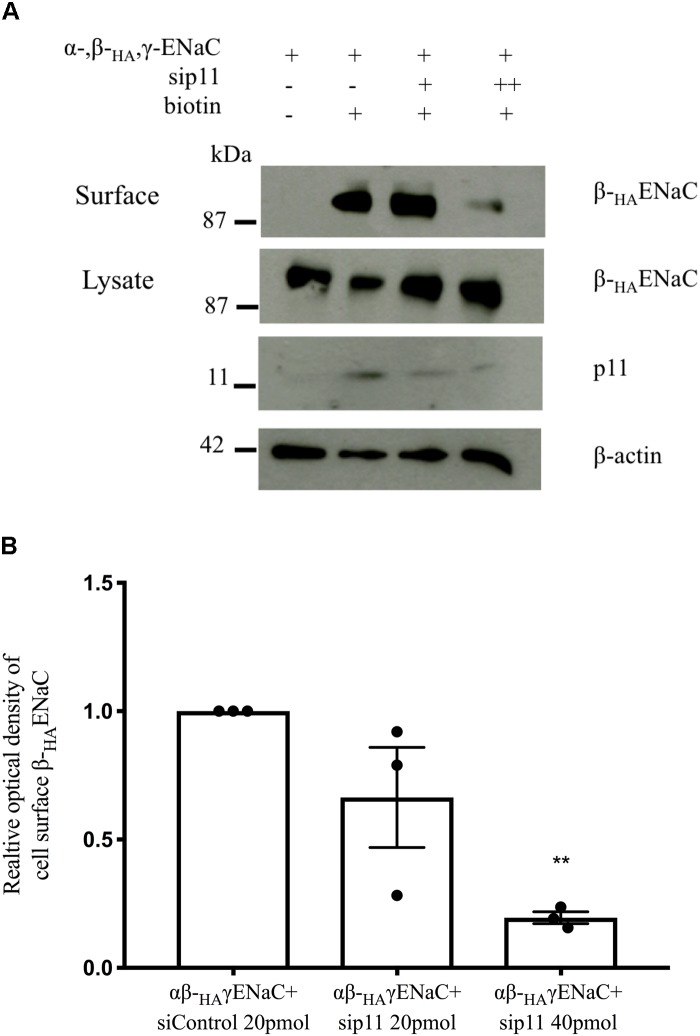
Knockdown of p11 reduces ENaC cell surface levels. FRT epithelia were cotransfected with plasmids encoding α-, β_HA_-, and γ ENaC (1 μg each) together with either control siRNA or increasing amounts of p11 siRNA (20 and 40 pmol). Cell surface proteins were labeled with Sulfo-NHS-LC-biotin and captured on Neutravidin beads after cell lysis. β_HA_ ENaC was detected in both the cell surface and whole cell lysate fractions. **(A)** Representative immunoblot of surface β_HA_ ENaC. **(B)** Pooled results show a significant decrease in the cell surface population of β_HA_ ENaC with 40 pmol sip11, *n* = 3, ^∗∗^*p* < 0.01 by one-way ANOVA followed by Bonferroni’s post-test.

To confirm and extend the observation that overexpression of p11 increases ENaC current in oocytes, we used a model mammalian epithelia, FRT epithelia, to test the effect of reduced p11 levels on ENaC current. Plasmids encoding the α-, β-, and γ ENaC subunits were introduced into FRT epithelia by transfection together with siRNA targeting p11 (si-p11), or a control siRNA (si-control). Figure 6A shows that epithelia expressing α-, β-, and γ- ENaC generated an amiloride-sensitive short-circuit current. In the presence of si-p11 the amiloride-sensitive current was reduced compared to si-control; and pooled data (Figure 6B) showed a significant reduction in ENaC current when p11 protein level was reduced (*n* = 6–7, *p* < 0.01). These results support the hypothesis that p11 is required for efficient delivery of ENaC to the cell surface but could also represent changes in ENaC endocytosis, open probability or proteolytic cleavage. Western blot analysis confirmed that si-p11 reduced p11 protein level in FRT cells by an average of 75% (*n* = 6–7, *p* < 0.01, also see Figure 7A for representative western blot showing p11 knockdown).

### Knockdown of p11 Reduces ENaC Cell Surface Population in FRT Cells

To further test the hypothesis that p11 regulates the cell surface population of ENaC we carried out cell surface protein biotinylation assays. FRT cells were transfected with plasmids encoding α-, β_HA_-, and γ ENaC together with equal amounts of si-control, or si-p11 (20 or 40 pmol). After 24 h, the cells were incubated with cell-impermeable sulfo-NHS-LC-biotin to label proteins at the cell surface. After cell lysis, biotinylated proteins were captured on Neutravidin^TM^ beads while the supernatant was retained as the cytosolic fraction. Figure 7A shows successful detection of β_HA_ ENaC in the cell surface and cytosolic fractions. The amount of β_HA_ ENaC at the cell surface was significantly reduced (*p* < 0.01, *n* = 3) in the presence of si-p11 (40 pmol) indicating that p11 alters the cell surface population of ENaC (Figure 7A,B and Supplementary Figure S5).

## Discussion

Epithelial Na^+^ channel channel function in CCD cells is subject to stringent regulation by a diversity of trafficking and signaling proteins to fine tune the amount of Na^+^ absorbed by the kidney. In this study we have shown that p11 can regulate ENaC channel function causing an increase in Na^+^ current (Figure 5, 6) through altering the cell surface population of ENaC (Figure 7). Additionally, p11 was found to physically bind to ENaC (Figure 1, 2), an interaction that may be required to generate the observed functional effects at the plasma membrane. We also demonstrated using mass spectrometry that the p11-ENaC complex associated with other proteins involved in cell trafficking (Table 1) suggesting that p11 may play an important role as a molecular hub controlling the number of ENaC channels at the membrane. Furthermore, the p11 protein is endogenously expressed in mouse CCD cells although its expression is not regulated by either short- or long-term exposure to aldosterone (Figure 4). Altogether, these results indicate that p11 plays an important regulatory role in stimulating ENaC channel function, however, more work is required to establish the mechanism of p11 regulation of ENaC. Models include p11 promoting exocytosis of ENaC, or p11 directly activating ENaC already at the cell surface.

Many of the well-characterized protein–protein interactions that occur between ENaC and cytosolic proteins serve to regulate the distribution of ENaC between intracellular compartments and the plasma membrane. Endocytosis of ENaC is perhaps the best understood of these pathways where members of the Nedd4 family bind to ENaC channel subunits to promote ENaC ubiquitination and subsequent channel internalization and degradation (Staub et al., 1997; McDonald et al., 2002). This pathway is further modulated by protein interactions with SGK1 (Snyder et al., 2002), 14-3-3 (Ichimura et al., 2005), GILZ1, Raf-1 (Soundararajan et al., 2009), and COMMDs (Ke et al., 2010). More recent studies highlight the importance of the recycling trafficking pathway in upregulating ENaC at the plasma membrane following endocytosis with roles for deubiquitinating enzymes (Butterworth et al., 2007; Fakitsas et al., 2007), Rab11b (Butterworth et al., 2012), and ankyrin G (Klemens et al., 2017).

In contrast, less is known about the cytosolic proteins controlling how ENaC is delivered to the plasma membrane via the exocytic pathway. In the present study we have demonstrated that p11, a protein with a well-defined role in directing vesicular trafficking along the exocytic pathway (Rescher and Gerke, 2008; Umbrecht-Jenck et al., 2010), interacts with all three ENaC subunits. This suggests that p11 binds to ENaC to promote its exocytic delivery to the plasma membrane and this is supported by our electrophysiology results where p11 overexpression caused an increase in ENaC specific current, and knockdown of p11 reduced ENaC specific current. This is also in agreement with other studies where p11 has been shown to bind to the Na_V_1.8 (Okuse et al., 2002), ASIC1a (Donier et al., 2005), TRPV5 and TRV6 (van de Graaf et al., 2003) channels resulting in an increased channel expression at the membrane.

**FIGURE 8:**
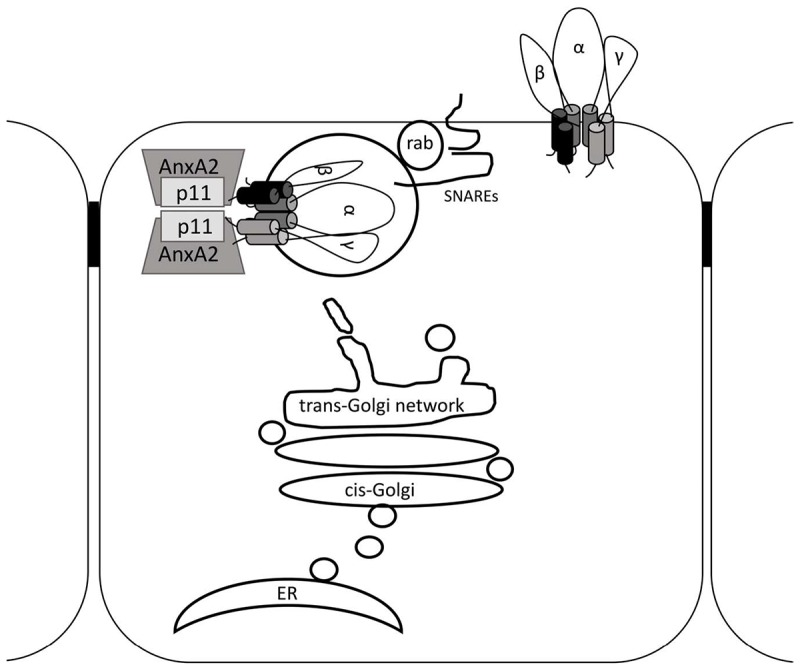
Schematic for p11 in ENaC trafficking. ENaC is translated at the endoplasmic reticulum (ER) and is modified by glycosylation, folded and assembled in the ER. ENaC is then moved to the *cis*-Golgi and emerges from the *trans*-Golgi network (TGN) in vesicles or tubules. The Annexin II-p11 tetramer may associate directly with vesicular ENaC and assist with apical targeting and tethering. At the apical membrane rab, v-SNARE, and t-SNARE molecules in the vesicle and apical membrane respectively will promote vesicle fusion, delivering ENaC to the apical cell surface. Not to scale.

The interaction between p11 and ENaC could take place at the *trans* Golgi network (TGN) where p11 in complex with annexin II could sequester ENaC to lipid rafts. Indeed, our results here show that annexin II is endogenously co-expressed with p11 in CCD cells. Moreover, a role for the p11-annexin II complex has been described for apical membrane delivery, in lipid rafts, of sucrase-isomaltase (Jacob et al., 2004) and the NKCC2 transporter (Dathe et al., 2014) in intestinal and renal epithelial cells respectively. Lipid rafts are also involved in the apical delivery of ENaC in CCD cells (Hill et al., 2007). Alternatively, p11 could act as a molecular tether linking ENaC containing vesicles to the cytosolic face of the plasma membrane before SNARE-mediated fusion. This is supported by observations that p11 is localized to the SNARE containing microdomains of exocytic sites on the cytoplasmic face of the plasma membrane, and that p11 interacts with the v-SNARE VAMP2 (Umbrecht-Jenck et al., 2010). Furthermore, ENaC interactions with several SNAREs are involved in insertion of the channel at the plasma membrane (Condliffe et al., 2003; Saxena et al., 2006). Overall, our results together with previous studies support a role for p11 in the exocytic delivery of ENaC to the plasma membrane (see Figure 8). Future studies investigating a potential link between SNAREs, p11 and ENaC in CCD cells will help to clarify whether the p11-ENaC interactions shown here occur at an early or late stage along the exocytic pathway.

Our mass spectroscopy data confirmed novel interactions between the p11-ENaC complex and other trafficking proteins: annexin A6 and HSP90. Annexin A6 is associated with multiple membrane proteins during their trafficking (Grewal et al., 2016; Enrich et al., 2017), while HSP90 is best known for its chaperone role in the secretory pathway (Schopf et al., 2017). It is not clear why ENaC or p11 peptides were not detected in our mass spectroscopy experiments, as they were confirmed to be present after the two rounds of co-immunoprecipitation. Although we might have expected annexin II or other ENaC trafficking proteins to be detected (e.g., AS160, Rab11b, or PKD) it is possible that the ENaC-p11 complex masks binding sites for these proteins preventing their co-precipitation.

In addition to targeting proteins that regulate ENaC endocytosis, aldosterone also has an effect on regulatory proteins in the exocytic pathway. Expression of the Rab-GAP AS160 in CCD cells is increased by aldosterone and aldosterone signaling phosphorylates AS160 to permit forward trafficking of ENaC to the apical membrane (Liang et al., 2010). Also, PKD1 localizes to the TGN in CCD cells where aldosterone treatment induces an interaction between PKD1 and PI4KIIIb and rapid translocation of ENaC to the apical membrane (Dooley et al., 2013). In contrast, our results here suggest that p11 expression is not modulated by aldosterone. Although not significant, there did appear to be a trend toward a decrease in p11 expression when full serum was removed which recovered with exposure to aldosterone over 24 h. Perhaps more sensitive methods for quantifying p11 expression may reveal a significant effect. Therefore, our results do support a role for p11 in promoting ENaC trafficking in the constitutive secretory pathway, but at present we do not have evidence for p11 being involved in the fine-tuning of ENaC exocytosis. However, it is possible that p11 expression and function may not be regulated by aldosterone but by other hormones such as vasopressin that target both p11 and ENaC. This is supported by a recent study by Dathe et al. (2014) who demonstrated that the NKCC2 transporter co-localizes and interacts with p11 and annexin II in renal epithelial cells and that vasopressin stimulates phosphorylation of annexin II to promote its trafficking to the apical membrane (Dathe et al., 2014). Further work will need to be carried out to test modulation of p11 expression by other regulators such as EGF, IGF and insulin.

The stimulatory effects of p11 on ENaC current could also be caused by p11 directly stimulating channels already resident at the membrane. In this scenario, p11 binding to cytosolic domains of ENaC channel subunits at the membrane may alter the gating of the channel to bring about the observed increase in Na^+^ current observed with p11 co-expression, see Figure 8. This is supported by a prior study of interactions between p11 and an apical membrane Cl^-^ channel in epithelial cells. Stimulating cAMP signaling in human bronchial epithelial cells caused an increase in the amount of p11-annexin II in complex with cell surface CFTR and a corresponding increase in CFTR Cl^-^ current (Borthwick et al., 2007). Therefore, interactions of p11 with ion channels may not only be important for trafficking channels to the membrane but p11 may remain bound to the channel at the membrane to influence channel activity. Such a dual effect of cytosolic protein interactions with ENaC and other channels has been described previously. Syntaxin1A has been shown to modulate both ENaC channel gating and the number of channels delivered to the membrane (Condliffe et al., 2004). Also, TRIP8 interacts with the C-terminal region of HCN1 channels to alter channel gating and differentially influence HCN1 trafficking to the membrane in a TRIP8 isoform dependent manner (Lewis et al., 2009). Therefore, the stimulatory effect of p11 on Na^+^ current that we have described here could be due to a combination of an increased ENaC population and due to p11 modifying how ENaC gates at the membrane.

In summary, our study has described a novel interaction between p11 and ENaC that is associated with increased channel function. Given the well-characterized role of p11 in the exocytic pathway, we propose that cytosolic interactions with ENaC promote the forward trafficking of ENaC to the membrane to cause the increased Na^+^ current although additional effects on ENaC channel activity are also possible. Although p11 expression in CCD cells did not appear to be modulated by aldosterone, further investigation into other hormonal pathways that control renal Na^+^ reabsorption may reveal a regulatory effect on p11 function. Overall, our results support a role for p11 in regulating ENaC functional activity furthering our understanding of the cellular pathways involved in Na^+^ homeostasis.

## Author Contributions

NI, TC, RM, NA, and SC performed the experiments. NI, TC, RM, FM, and SC analyzed the data. NI, TC, FM, and SC designed the experiments and wrote the manuscript. TC, NI, RM, NA, and FM approved the final manuscript.

## Conflict of Interest Statement

The authors declare that the research was conducted in the absence of any commercial or financial relationships that could be construed as a potential conflict of interest.
