# Supplementary figures and images for: Annexin II Light Chain p11 Interacts With ENaC to Increase Functional Activity at the Membrane
Source: Front Physiol. 2019 Feb 8;10:7. doi: 10.3389/fphys.2019.00007 (PMC6375906; doi:10.3389/fphys.2019.00007)

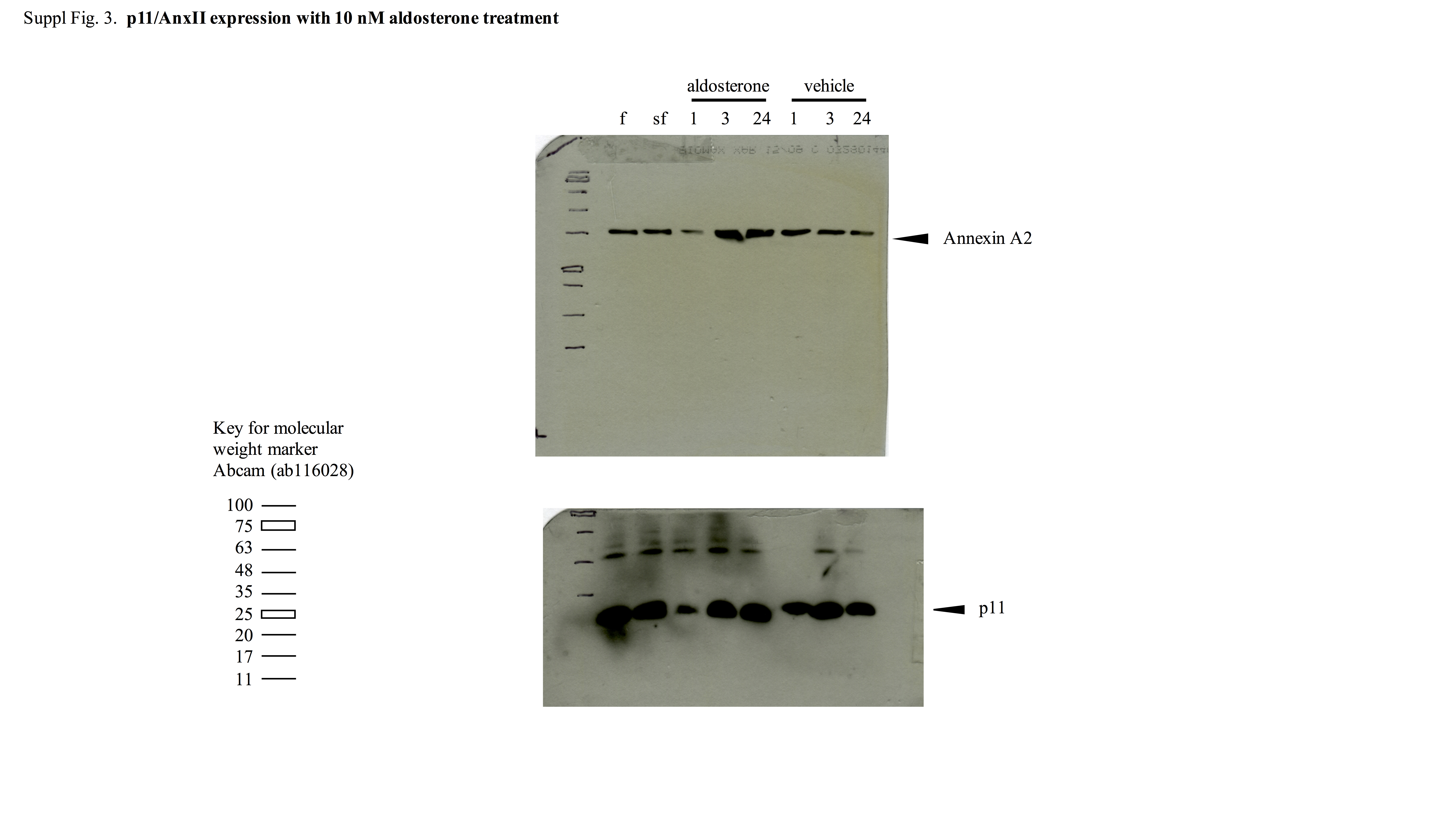

Supplement: Supplementary file 3 [file Image_3.jpg]
