# Supplementary material for: Annexin II Light Chain p11 Interacts With ENaC to Increase Functional Activity at the Membrane
Source: Front Physiol. 2019 Feb 8;10:7. doi: 10.3389/fphys.2019.00007 (PMC6375906; doi:10.3389/fphys.2019.00007)
Supplement: Supplementary file 6 [file Table_1.DOCX]

**Supplementary table 1**

**List of all proteins identified by mass spectrometry sequencing of material precipitated with the p11-ENaC complex and associated Mascot score**

| **Accession** | **Description** | **Mascot**  **Score** |
| --- | --- | --- |
| P09874 | Poly [ADP-ribose] polymerase 1 OS=Homo sapiens GN=PARP1 PE=1 SV=4 - [PARP1_HUMAN] | 360.07 |
| P22626-2 | Isoform A2 of Heterogeneous nuclear ribonucleoproteins A2/B1 OS=Homo sapiens GN=HNRNPA2B1 - [ROA2_HUMAN] | 222.17 |
| P23246 | Splicing factor, proline- and glutamine-rich OS=Homo sapiens GN=SFPQ PE=1 SV=2 - [SFPQ_HUMAN] | 219.53 |
| P12956 | X-ray repair cross-complementing protein 6 OS=Homo sapiens GN=XRCC6 PE=1 SV=2 - [XRCC6_HUMAN] | 209.74 |
| O75367-3 | Isoform 3 of Core histone macro-H2A.1 OS=Homo sapiens GN=H2AFY - [H2AY_HUMAN] | 179.48 |
| P06748 | Nucleophosmin OS=Homo sapiens GN=NPM1 PE=1 SV=2 - [NPM_HUMAN] | 178.62 |
| Q9NR30 | Nucleolar RNA helicase 2 OS=Homo sapiens GN=DDX21 PE=1 SV=5 - [DDX21_HUMAN] | 176.84 |
| P11142 | Heat shock cognate 71 kDa protein OS=Homo sapiens GN=HSPA8 PE=1 SV=1 - [HSP7C_HUMAN] | 157.87 |
| P07437 | Tubulin beta chain OS=Homo sapiens GN=TUBB PE=1 SV=2 - [TBB5_HUMAN] | 151.91 |
| P08107 | Heat shock 70 kDa protein 1A/1B OS=Homo sapiens GN=HSPA1A PE=1 SV=5 - [HSP71_HUMAN] | 151.46 |
| Q00839-2 | Isoform Short of Heterogeneous nuclear ribonucleoprotein U OS=Homo sapiens GN=HNRNPU - [HNRPU_HUMAN] | 151.20 |
| P68371 | Tubulin beta-4B chain OS=Homo sapiens GN=TUBB4B PE=1 SV=1 - [TBB4B_HUMAN] | 149.74 |
| P19338 | Nucleolin OS=Homo sapiens GN=NCL PE=1 SV=3 - [NUCL_HUMAN] | 146.39 |
| P38646 | Stress-70 protein, mitochondrial OS=Homo sapiens GN=HSPA9 PE=1 SV=2 - [GRP75_HUMAN] | 143.76 |
| P38159 | RNA-binding motif protein, X chromosome OS=Homo sapiens GN=RBMX PE=1 SV=3 - [RBMX_HUMAN] | 135.46 |
| Q13885 | Tubulin beta-2A chain OS=Homo sapiens GN=TUBB2A PE=1 SV=1 - [TBB2A_HUMAN] | 134.90 |
| P62805 | Histone H4 OS=Homo sapiens GN=HIST1H4A PE=1 SV=2 - [H4_HUMAN] | 133.51 |
| P13010 | X-ray repair cross-complementing protein 5 OS=Homo sapiens GN=XRCC5 PE=1 SV=3 - [XRCC5_HUMAN] | 119.96 |
| Q9BQE3 | Tubulin alpha-1C chain OS=Homo sapiens GN=TUBA1C PE=1 SV=1 - [TBA1C_HUMAN] | 119.45 |
| P38919 | Eukaryotic initiation factor 4A-III OS=Homo sapiens GN=EIF4A3 PE=1 SV=4 - [IF4A3_HUMAN] | 116.01 |
| P68104 | Elongation factor 1-alpha 1 OS=Homo sapiens GN=EEF1A1 PE=1 SV=1 - [EF1A1_HUMAN] | 105.45 |
| P16403 | Histone H1.2 OS=Homo sapiens GN=HIST1H1C PE=1 SV=2 - [H12_HUMAN] | 102.84 |
| P10412 | Histone H1.4 OS=Homo sapiens GN=HIST1H1E PE=1 SV=2 - [H14_HUMAN] | 100.22 |
| P52272-2 | Isoform 2 of Heterogeneous nuclear ribonucleoprotein M OS=Homo sapiens GN=HNRNPM - [HNRPM_HUMAN] | 95.57 |
| Q96PK6 | RNA-binding protein 14 OS=Homo sapiens GN=RBM14 PE=1 SV=2 - [RBM14_HUMAN] | 86.83 |
| Q93077 | Histone H2A type 1-C OS=Homo sapiens GN=HIST1H2AC PE=1 SV=3 - [H2A1C_HUMAN] | 86.61 |
| P08238 | Heat shock protein HSP 90-beta OS=Homo sapiens GN=HSP90AB1 PE=1 SV=4 - [HS90B_HUMAN] | 78.99 |
| Q96KK5 | Histone H2A type 1-H OS=Homo sapiens GN=HIST1H2AH PE=1 SV=3 - [H2A1H_HUMAN] | 78.46 |
| Q13509 | Tubulin beta-3 chain OS=Homo sapiens GN=TUBB3 PE=1 SV=2 - [TBB3_HUMAN] | 76.66 |
| Q9H0S4-2 | Isoform 2 of Probable ATP-dependent RNA helicase DDX47 OS=Homo sapiens GN=DDX47 - [DDX47_HUMAN] | 76.53 |
| P60709 | Actin, cytoplasmic 1 OS=Homo sapiens GN=ACTB PE=1 SV=1 - [ACTB_HUMAN] | 72.72 |
| P41091 | Eukaryotic translation initiation factor 2 subunit 3 OS=Homo sapiens GN=EIF2S3 PE=1 SV=3 - [IF2G_HUMAN] | 64.55 |
| P0C0S5 | Histone H2A.Z OS=Homo sapiens GN=H2AFZ PE=1 SV=2 - [H2AZ_HUMAN] | 45.43 |
| P11021 | 78 kDa glucose-regulated protein OS=Homo sapiens GN=HSPA5 PE=1 SV=2 - [GRP78_HUMAN] | 43.93 |
| O76021 | Ribosomal L1 domain-containing protein 1 OS=Homo sapiens GN=RSL1D1 PE=1 SV=3 - [RL1D1_HUMAN] | 43.16 |
| H0Y4X3 | RNA-binding protein 39 (Fragment) OS=Homo sapiens GN=RBM39 PE=1 SV=2 - [H0Y4X3_HUMAN] | 38.84 |
| P0C0S5 | Histone H2A.Z OS=Homo sapiens GN=H2AFZ PE=1 SV=2 - [H2AZ_HUMAN] | 38.24 |
| P62805 | Histone H4 OS=Homo sapiens GN=HIST1H4A PE=1 SV=2 - [H4_HUMAN] | 34.68 |
| P35250-2 | Isoform 2 of Replication factor C subunit 2 OS=Homo sapiens GN=RFC2 - [RFC2_HUMAN] | 26.56 |
| Q99496 | E3 ubiquitin-protein ligase RING2 OS=Homo sapiens GN=RNF2 PE=1 SV=1 - [RING2_HUMAN] | 24.52 |
| O00425 | Insulin-like growth factor 2 mRNA-binding protein 3 OS=Homo sapiens GN=IGF2BP3 PE=1 SV=2 - [IF2B3_HUMAN] | 24.42 |
| F5H265 | Polyubiquitin-C (Fragment) OS=Homo sapiens GN=UBC PE=4 SV=1 - [F5H265_HUMAN] | 21.15 |
| P05387 | 60S acidic ribosomal protein P2 OS=Homo sapiens GN=RPLP2 PE=1 SV=1 - [RLA2_HUMAN] | 18.99 |
| F5H265 | Polyubiquitin-C (Fragment) OS=Homo sapiens GN=UBC PE=4 SV=1 - [F5H265_HUMAN] | 18.59 |
| G3V5X6 | Heterogeneous nuclear ribonucleoproteins C1/C2 (Fragment) OS=Homo sapiens GN=HNRNPC PE=1 SV=1 - [G3V5X6_HUMAN] | 13.01 |
| F5GX33 | Calcium-binding mitochondrial carrier protein Aralar2 OS=Homo sapiens GN=SLC25A13 PE=1 SV=1 - [F5GX33_HUMAN] | 12.80 |
| S4R402 | Nucleolar and coiled-body phosphoprotein 1 OS=Homo sapiens GN=NOLC1 PE=1 SV=1 - [S4R402_HUMAN] | 12.05 |
| F8VRD2 | Rac GTPase-activating protein 1 (Fragment) OS=Homo sapiens GN=RACGAP1 PE=1 SV=2 - [F8VRD2_HUMAN] | 10.97 |
| Q86TV8 | Full-length cDNA clone CS0DI031YK16 of Placenta of Homo sapiens (human) OS=Homo sapiens GN=TDP1 PE=1 SV=1 - [Q86TV8_HUMAN] | 10.92 |
| Q71U36-2 | Isoform 2 of Tubulin alpha-1A chain OS=Homo sapiens GN=TUBA1A - [TBA1A_HUMAN] | 10.62 |
| P23527 | Histone H2B type 1-O OS=Homo sapiens GN=HIST1H2BO PE=1 SV=3 - [H2B1O_HUMAN] | 10.43 |
| I3L3P7 | 40S ribosomal protein S15a OS=Homo sapiens GN=RPS15A PE=1 SV=1 - [I3L3P7_HUMAN] | 8.75 |
| F8WF32 | Dolichyl-diphosphooligosaccharide--protein glycosyltransferase subunit 1 OS=Homo sapiens GN=RPN1 PE=1 SV=1 - [F8WF32_HUMAN] | 8.63 |
| O60814 | Histone H2B type 1-K OS=Homo sapiens GN=HIST1H2BK PE=1 SV=3 - [H2B1K_HUMAN] | 5.85 |
| J3QSA3 | Ubiquitin (Fragment) OS=Homo sapiens GN=UBB PE=4 SV=1 - [J3QSA3_HUMAN] | 5.82 |
| J3KSR7 | U3 small nucleolar RNA-associated protein 18 homolog (Fragment) OS=Homo sapiens GN=UTP18 PE=4 SV=1 - [J3KSR7_HUMAN] | 5.79 |
| H3BRN7 | Uncharacterized protein OS=Homo sapiens PE=4 SV=1 - [H3BRN7_HUMAN] | 5.41 |
| Q9Y3B4 | Splicing factor 3B subunit 6 OS=Homo sapiens GN=SF3B6 PE=1 SV=1 - [SF3B6_HUMAN] | 4.90 |
| M0R1I1 | Tubulin beta-4A chain (Fragment) OS=Homo sapiens GN=TUBB4A PE=3 SV=1 - [M0R1I1_HUMAN] | 4.50 |
| P05387 | 60S acidic ribosomal protein P2 OS=Homo sapiens GN=RPLP2 PE=1 SV=1 - [RLA2_HUMAN] | 3.65 |
| E5RIU8 | Annexin A6 OS=Homo sapiens GN=ANXA6 PE=1 SV=2 - [E5RIU8_HUMAN] | 3.51 |
